# A Case Report on a Rare Type of Lymphoma: Angioimmunoblastic T-cell Lymphoma

**DOI:** 10.7759/cureus.61670

**Published:** 2024-06-04

**Authors:** Andreia S Machado, Ana Catarina B Marques, Antony Soares Dionísio, Beatriz S Ferreira, Tiago M Marques

**Affiliations:** 1 Internal Medicine Department, Unidade Local de Saúde Ocidental, Hospital São Francisco Xavier, Lisbon, PRT; 2 Pathology and Laboratory Department, Unidade Local de Saúde de Santa Maria, Hospital Santa Maria, Lisbon, PRT; 3 Infectious Disease Department, Unidade Local de Saúde de Santa Maria, Hospital Santa Maria, Lisbon, PRT

**Keywords:** non-hodgkin lymphoma, angioimmunoblastic t-cell lymphoma, eosinophilia, lymphadenopathy, peripheral t-cell lymphoma

## Abstract

Angioimmunoblastic T-cell lymphoma (AITL) is a rare type of non-Hodgkin lymphoma (NHL). We present a case of a 60-year-old female who attended the emergency department (ED) with fatigue, recurrent fever, weight loss, and adenopathy for six months. Laboratory findings showed anemia, lymphocytosis, eosinophilia, thrombocytosis, cholestasis, hypoproteinemia, and hypoalbuminemia. Abdominopelvic computed tomography (CT) revealed multiple adenopathies. A lymph node biopsy yielded inconclusive results in the outpatient clinic. Later, during admission, the patient underwent a positron emission tomography-computed tomography (PET-CT), revealing a cervical adenopathy cluster that was excised en bloc. Histology confirmed the diagnosis of AITL. The medical team initiated chemotherapy but opted for exclusive symptomatic treatment due to disease progression. The patient died six months after diagnosis. The fluctuating and nonspecific presentation of AITL can hinder and delay definitive diagnosis, therefore impacting treatment and prognosis.

## Introduction

Angioimmunoblastic T-cell lymphoma (AITL) accounts for 1%-2% of non-Hodgkin lymphomas (NHL) and 18%-36% of the peripheral T-cell lymphoma (PTCL) subtype [1-3]. Phenotypically, it manifests as a follicular helper T-cell lymphoma, contributing to its heterogeneous and nonspecific presentation and posing a notable diagnostic challenge [4]. Definitive diagnosis requires biopsy and histopathological evaluation, including morphological, immunophenotypic, and molecular assessments [5]. Despite initially exhibiting a favorable response to chemotherapy, most patients eventually experience disease progression [3]. With this clinical case, we aim to raise awareness of this diagnosis due to its rarity and high mortality associated with delayed diagnosis.

## Case presentation

**Figure 1:**
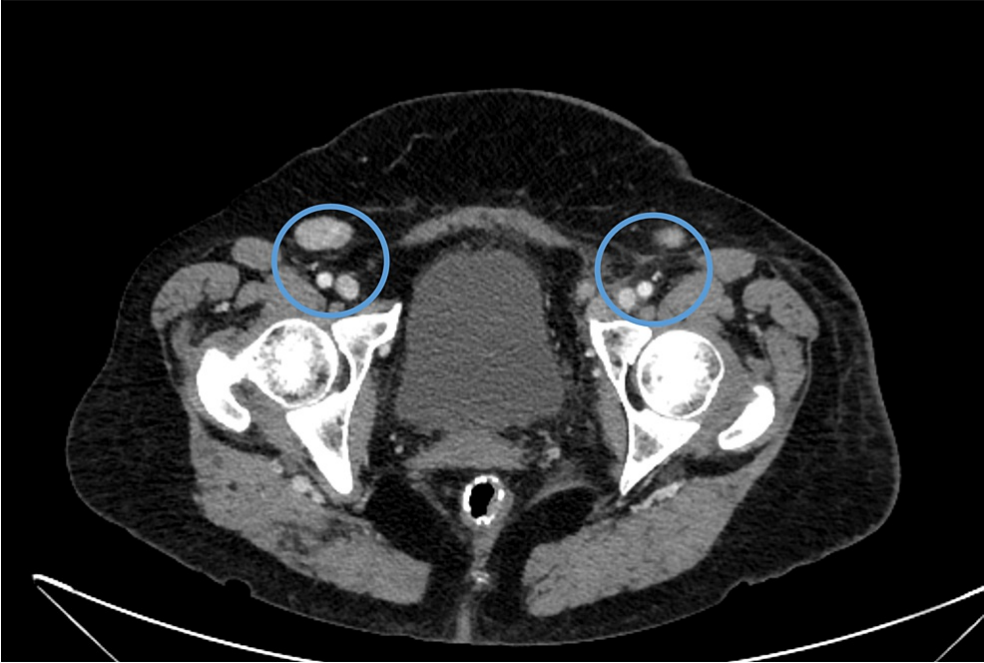
Enlarged lymph nodes in the pelvic CT scan CT: computed tomography

A 60-year-old female requiring assistance in her daily activities, with a history of osteogenesis imperfecta, presented to the emergency department (ED) with a six-month history of fatigue for moderate exertion with progressively worsening, daily febrile spikes reaching a maximum temperature of 38.5°C; weight loss exceeding 10%; and palpable axillary, inguinal, and cervical lymphadenopathy. Laboratory findings showed anemia, leukocytosis, thrombocytopenia, inflammatory markers, and lactate dehydrogenase (LDH) levels (Table 1). A computed tomography (CT) scan of the body revealed infra-diaphragmatic lymphadenopathy (Figure 1).

**Table 1:** Laboratory findings at first presentation to the emergency department (ED) and during admission

Laboratory parameter	Normal range	First episode in ED	Hospitalization one month after the ED episode
Hemoglobin (g/dL)	12-15.3	10.7	7.9
Mean corpuscular volume (fL)	80-96.1	89.6	90.5
Mean corpuscular hemoglobin (pg)	27.3-33.7	30	31
Reticulocyte index (%)	<15.6	21.6	19.5
Haptoglobin (mg/dL)	30-200	-	40
Iron (μg/dL)	33-193	-	25
Ferritin (ng/mL)	30-340	2356	3891
Total iron-binding capacity (μg/dL)	250-425	-	190
Transferrin saturation (%)	20-45	-	39
Folate (mmol/L)	10-42	-	21
Vitamin B12 (pmol/L)	141-489	-	644
Beta-2 microglobulin (mg/dL)	1.09-2.53	-	4.79
White blood cells (×10^9^/L)	4.0-11.0	14.8	20.4
Neutrophils (×10^9^/L)	1.9-7.5	4.4	17.0
Lymphocytes (×10^9^/L)	1.0-4.8	0.64	2.2
Eosinophils (×10^9^/L)	0-0.5	0.32	4.2
Platelets (×10^9^/L)	150-450	254	83
Erythrocyte sedimentation rate (mm/hour)	<20	69	80
C-reactive protein (mg/dL)	<0.5	2.76	5.74
Procalcitonin (ng/dL)	<0.5	0.27	0.5
Lactate dehydrogenase (U/L)	100-250	529	1379
Alanine aminotransferase (U/L)	0-33	57	60
Gamma-glutamyl transferase (U/L)	0-40	92	183
Alkaline phosphatase (U/L)	35-105	220	497
Total protein (g/dL)	6.6-8.7	6.4	5
Albumin (g/dL)	3.5-5.2	3.0	2.8
International normalized ratio	1	1.06	1.4
Urea (mg/dL)	16-49	50	95
Creatinine (mg/dL)	0.5-0.9	0.6	0.48
Sodium (mEq/L)	136-145	139	133
Potassium (mEq/L)	3.5-5.1	4.96	5.0
Calcium (mg/dL)	8.4-10.2	8.7	8.3

As an outpatient, she underwent an incisional biopsy of an inguinal lymph node with inconclusive results. Given the suspicion of lymphoid neoplasm, the patient began corticosteroid therapy while awaiting scheduling for positron emission tomography-computed tomography (PET-CT).

**Figure 2:**
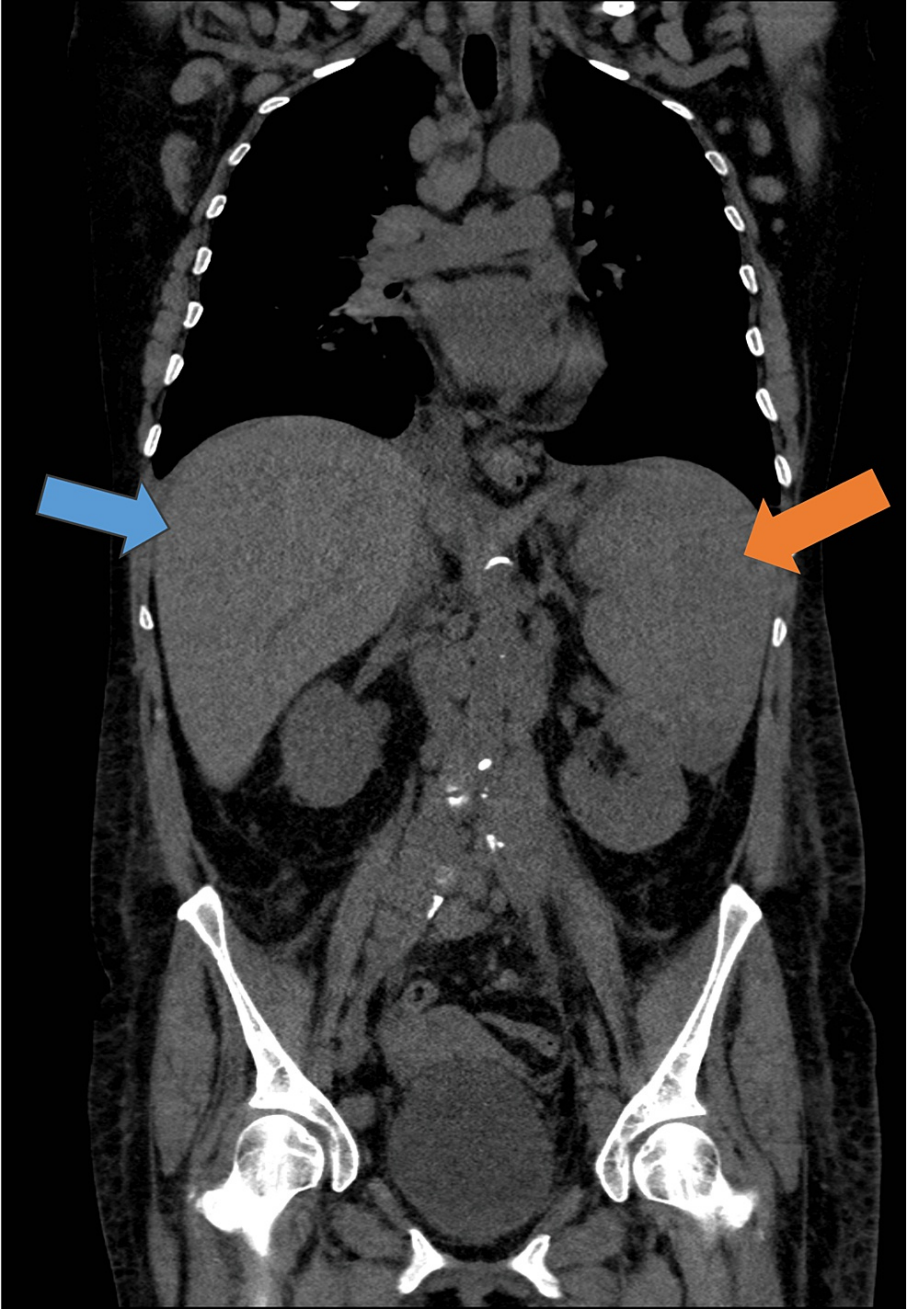
Computed tomography Blue arrow, hepatomegaly; orange arrow, splenomegaly

Two months after her first visit to the ED, she began experiencing dyspnea, left pleuritic chest pain, and worsening dry cough while maintaining the previous fever pattern. A subsequent CT scan showed an enlargement in the size and number of adenopathies and hepatosplenomegaly (Figure 2). Because of the urgent need to elucidate the clinical presentation and respiratory failure, the patient was admitted. She underwent an incisional biopsy of a cervical lymph node, revealing eosinophilic infiltration, areas of necrosis, granulomas, activated B-cells, and inconclusive T-cell clonality.

Considering the prolonged fever of unknown origin, autoimmune and infectious etiologies were ruled out through analytical, serological, and microbiological studies (Table 2 and Table 3). Hematologic analysis revealed hypervitaminosis B12 and elevated beta-2 microglobulin levels (Table 1).

**Table 2:** Infectious disease panel performed during the patient's hospitalization

Infectious agents	First episode in ED	Hospitalization one month after the ED episode
Hepatitis B surface antigen	-	Negative
Hepatitis B surface (HBs) antibody	-	Positive
Hepatitis B core antibody	-	Negative
Hepatitis A antibody	-	Negative
Hepatitis C antibody	-	Negative
Antibodies against HIV-1 and HIV-2	Negative	-
Epstein-Barr IgM viral capsid antibody	Negative	-
Epstein-Barr IgG viral capsid antibody	Positive	-
Cytomegalovirus IgM antibody	Negative	-
Cytomegalovirus IgG antibody	Positive	-
Parvovirus B19	-	Negative
Enterovirus	-	Negative
Brucella IgG antibody	-	Negative
Brucella IgM antibody	-	Negative
*Bartonella* spp.	-	Negative
Rickettsia conorii	-	Negative
Leishmania	-	Negative
*Legionella pneumophila* urinary antigen	Negative	-
*Plasmodium* spp.	-	Negative
Interferon gamma release assay (IGRA)	-	Negative
Blood, urine, and sputum cultures for bacteria, fungi, and mycobacteria	Negative	Negative
*Strongyloides* IgG antibody	-	Negative

**Table 3:** Autoimmune panel performed during hospitalization Sm, Smith; RNP, ribonucleoprotein; LKM-1, liver/kidney microsome type 1; LC-1, liver cytosol antigen type 1; SLA/LP, soluble liver antigen/liver-pancreas; AMA, anti-mitochondrial; PML, promyelocytic leukemia; gp210, glycoprotein-210; c-ANCA, cytoplasmic antineutrophil cytoplasmic antibody; p-ANCA, perinuclear antineutrophil cytoplasmic antibody; dsDNA, double-stranded deoxyribonucleic acid

Autoimmune panel	Normal range	Hospitalization
Complement C3 (mg/dL)	90-180	121
Complement C4 (mg/dL)	10-40	56.4
Rheumatoid factor (IU/L)	<15	<9.19
Anti-cyclic citrullinated peptide antibodies (EU/mL)	<5	<0.1
Anti-nuclear antibodies	Negative	Negative
Anti-dsDNA antibodies	Negative	Negative
Anti-Ro/La/Sm/U1-RNP/Scl-70/Jo-1	Negative	Negative
Anti-hepatic antigen antibodies (LKM-1, LC-1, SLA/LP, AMA-M2, AMA-M2-3E, Sp 100, PML, and gp210)	Negative	Negative
Anti-Ro52 antibody	Negative	Negative
Antineutrophil cytoplasmic antibody (c-ANCA and p-ANCA)	Negative	Negative

**Figure 3:**
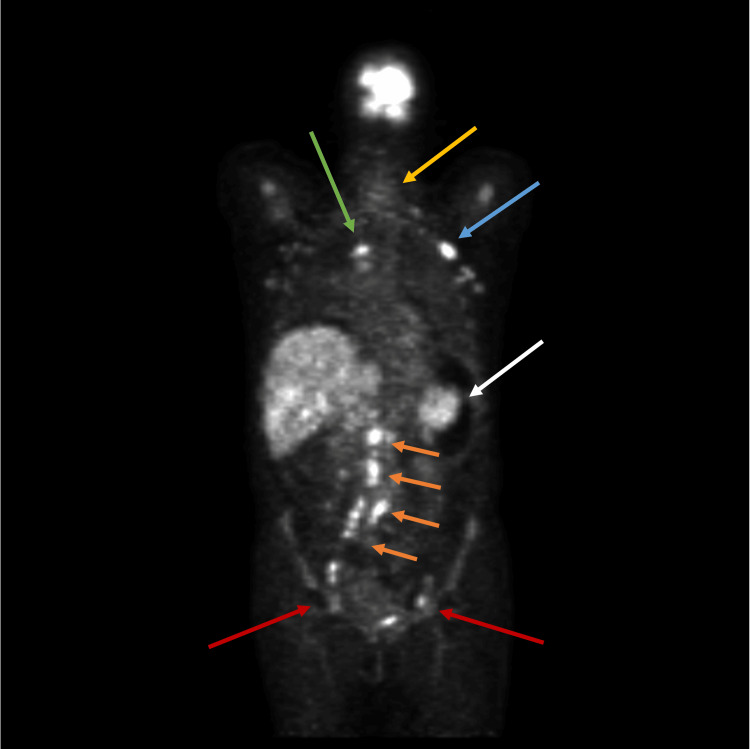
Positron emission tomography-computed tomography made during the patient's admission Red arrows, inguinal adenopathies; orange arrows, lombo-aortic adenopathies; white arrow, splenomegaly; blue arrow, axillary adenopathy; green arrow, mediastinal adenopathy; yellow arrow, cervical adenopathy cluster

A PET-CT scan revealed multiple supra- and infra-diaphragmatic adenopathies, bone marrow, and spleen avidly uptaking radioisotope (Figure 3). The definitive diagnosis was only achieved through a cervical lymph node biopsy, confirming angioimmunoblastic T-cell lymphoma, Ann Arbor IV, associated with Epstein-Barr virus infection. A cranioencephalic magnetic resonance imaging ruled out leptomeningeal invasion.

The medical team started a chemotherapy regimen with six cycles of cyclophosphamide, doxorubicin, vincristine, and prednisolone (CHOP). Initially, the medical team observed a favorable response in a CT scan with a reduction of the size of the lymph nodes, but after three months, after the beginning of chemotherapy, disease progression was documented in a follow-up PET-CT scan. After a multidisciplinary discussion and considering her current clinical status, the team decided that the patient was a candidate for exclusive symptomatic treatment. Death occurred six months after the initial diagnosis, in the context of a respiratory septic shock refractory to medical support.

## Discussion

AITL is a rare form of NHL and is often a rapidly progressive condition, with over 80% of patients diagnosed at an advanced stage [3]. It is more prevalent in patients over 60 years and in Europeans, as observed in the reported case [1,6].

Initially, researchers defined AITL as an abnormal immune reaction or atypical lymphoid hyperplasia. After the discovery of the underlying cytogenetic alterations, the World Health Organization defined it as a neoplasm of helper T-cells [7]. The microenvironment in AITL is marked by the overexpression of B-cells, dendritic cells, chemokines, and genes related to the extracellular matrix, vascular matrix, and helper T-cells. Despite extensive research, the specific risk factors for the development of AITL remain unidentified [4].

As seen in this case, the clinical presentation includes B symptoms in over 70% of cases and extranodal involvement in 21%-46% of cases, most observed in the spleen (31%-35%), liver (3.2%-26%), and bone marrow (18.6%). Cutaneous rash is documented in 13%-44% of patients, arthralgia in 16%, and ascites, edema, or pleural effusion in 14%-26%. High LDH (60%-66%), hypergammaglobulinemia (30%-50%), and eosinophilia (32%) are common and were present in this case. Around 10% of cases may find a positive autoimmune panel, and leukocytosis with lymphocytosis is rare but was found in this case [1,3,4].

In addition to a thorough clinical history and laboratory findings, imaging studies play a crucial role in identifying sites of increased disease activity, ensuring accurate biopsy sampling since it is a rare type of lymphoma, with a vast differential diagnosis, and depends on the experience of the pathologist. Despite being a well-defined disease, definitive diagnosis remains challenging due to neoplastic cells representing a minority of the infiltrate, and they are usually near the venules, so excisional biopsy is the best option [4]. In the beginning, the team tried to find the diagnosis with a needle core, but after the negative result, they tried an incisional biopsy with an inconclusive result. Probably, this happened because those kinds of biopsies do not get enough tissue to reach the diagnosis.

AITL management is like other PTCLs, with the current standard of care being a CHOP regimen [1]. Although initial responses to treatment are often positive, most cases relapse, as seen in this case. Numerous studies have tried etoposide plus CHOP, yielding promising results, but it has limited application in elderly patients due to its high toxicity.

Autologous hematopoietic stem cell transplantation may be an option, but the team knew that was going to be too aggressive and unviable in this patient with marked fragility [4].

Some prognostic factors have been identified, including age over 60, marked functional limitation, the involvement of multiple extranodal sites, mediastinal lymphadenopathy, anemia, and thrombocytopenia [1,4,6]. Our patient met four severity criteria, indicating a poorer prognosis, which led to the exclusion of alternative treatment strategies besides the CHOP regimen.

According to literature findings, AITL is related to a poor prognosis, featuring a five-year overall survival of 32%-44% and a progression-free survival of 25%-33%. Most patients succumb to disease progression (91%) or infectious complications (7%) [3].

Unfortunately, our patient was diagnosed too late, with several prognostic risk factors that may have contributed to a failure to respond to chemotherapy and subsequent septic shock due to immunosuppression, which led to her death six months after diagnosis.

## Conclusions

AITL is a rare form of lymphoma characterized by nonspecific and varied clinical-pathological features, which can mimic several other diagnoses. A definitive diagnosis depends on the experience of the pathologist and requires an excisional biopsy. Despite being a well-defined disease, the neoplastic cells represent a minority of the infiltrate. Most cases find the diagnosis in advanced stages, and available therapeutic options are of limited efficacy in the medium to long term.
